# Carbon Nanotube-Reinforced Thermotropic Liquid Crystal Polymer Nanocomposites

**DOI:** 10.3390/ma2041955

**Published:** 2009-11-26

**Authors:** Jun Young Kim

**Affiliations:** 1 Material Laboratory, Corporate R&D Center, Samsung SDI Co. Ltd., 575 Shin-dong, Yeongtong-gu, Suwon-si, Gyeonggi-do, 443-731, Korea; E-Mail: junykim74@hanmail.net; Tel.: +82-31-210-7103; Fax: +82-31-210-7374; 2 Department of Materials Science and Engineering, Massachusetts Institute of Technology, 77 Massachusetts Avenue, Cambridge, MA 02139, USA

**Keywords:** carbon nanotube, nanocomposites, thermotropic liquid crystal polymer

## Abstract

This paper focuses on the fabrication via simple melt blending of thermotropic liquid crystal polyester (TLCP) nanocomposites reinforced with a very small quantity of modified carbon nanotube (CNT) and the unique effects of the modified CNT on the physical properties of the nanocomposites. The thermal, mechanical, and rheological properties of modified CNT-reinforced TLCP nanocomposites are highly dependent on the uniform dispersion of CNT and the interactions between the CNT and TLCP, which can be enhanced by chemical modification of the CNT, providing a design guide of CNT-reinforced TLCP nanocomposites with great potential for industrial uses.

## 1. Introduction

Liquid-crystalline polymers (LCPs) have a phase of matter intermediate between the isotropic liquid and crystalline solid states termed as a mesophase or mesomorphic phase, and this LC state offers broad perspectives for various application materials that exhibit anisotropic but liquid properties [1]. In general, LCPs can be distinguished as thermotropic LCPs or lyotropic LCPs, depending on whether they are in a LC state in the melt or in solution. Lyotropic LCPs exhibit liquid crystallinity in the solution, being controlled by the characteristics and temperature of solvent and the concentration of polymer, and they cannot exhibit liquid crystallinity in the melt because they degrade before melting and show a phase transition through the addition or removal of solvents [2,3]. For thermotropic LCPs, the phase transition to LC state is induced by a thermal process, being controlled by the melt temperature and thermal history, and they form thermally activated mesogenic phases that extend from the crystal melting temperature up to the isotropic temperature [2,3].

Thermotropic LCPs, that are a class of the materials used to produce anisotropic melts of relatively low viscosity, have been extensively investigated because of their excellent mechanical properties, superior chemical resistance, low gas and liquid permeability, low controllable coefficient of thermal expansion, excellent dimensional stability under temperature or in severe environment, and easy processability with high precision by extrusion and injection molding [2,3,4,5]. Thermotropic LCPs with high strength and stiffness due to their rigid-rod- like molecules can be preferentially oriented to form fibrils under elongational or shear flow during melt processing, and oriented fibrous structures are developed in the extruded thermotropic LCPs, resulting in self-reinforcing characteristics [6,7,8]. For this reason, thermotropic LCPs have received considerable attention both in the neat state and as reinforcing fillers for thermoplastic polymers, and much research has been performed to date both to displace conventional thermoplastic polymers and to develop commercial applications of the thermotropic LCPs and their composites such a high performance engineering plastics or fibers [9,10,11,12].

Carbon nanotubes (CNTs) have attracted a great deal of scientific interest as advanced materials since they were first reported by Iijima [13]. In particular, the excellent electronic properties, thermal conductivity and mechanical strength of CNTs have created a high level of activity in materials research for practical applications in a broad range of industries such as hydrogen storage, lithium batteries, fuel cells, field-emitting-diodes, gas sensors, and polymer nanocomposites [14,15,16,17,18,19,20,21,22]. Furthermore, a number of efforts have been made to develop high performance polymeric materials based on CNTs, with the benefit of nanotechnology. Fundamental research progress on the potential applications of CNTs suggests that CNTs can be regarded as promising reinforcements in polymer nanocomposites due to the combination of their unique excellent properties with high aspect ratio and small size [23,24,25,26,27,28]. However, due to their high cost and limited availability, only a few practical applications have been realized to date in the various industrial fields.

Polymer nanocomposites that constitute a new class of the materials based on the reinforcement of polymers using nanofillers have attracted a great deal of interest in fields ranging from the purely scientific to industrial applications due to the remarkable improvements in the overall properties achieved at low filler loadings. For the fabrication of polymer nanocomposites containing CNTs, some major goals to realize the potential application of CNTs as nanoreinforcing fillers are: (a) homogeneous dispersion of the nanoutbes in the polymer matrix and (b) good interfacial adhesion between the nanotubes and the polymer matrix. Typically, CNTs tend to bundle together and to form agglomerations due to intrinsic van der Waals attractions between the individual tubes [24,25]. Weak interfacial bonding between CNTs and polymer matrix has limited efficient load transfer to polymers, playing a limited reinforcement role in the polymer nanocomposites [29,30,31]. The functionalization of CNTs, which can be considered as an effective method to achieve the uniform dispersion of CNTs and their compatibility with the polymers, can lead to the enhancement of the interfacial adhesion between CNTs and polymer matrix, thus improving physical properties of polymer nanocomposites containing CNTs [32,33,34]. In addition, one of major challenges for the fabrication of CNT/polymer nanocomposites is to optimize the processing operations for cheap manufacturing costs. Currently, four processing techniques are in common use to fabricate CNT/polymer nanocomposites [35,36,37,38,39,40]: direct mixing, solution method, *in situ* polymerization, and melt blending. Among these processing techniques, melt blending process has been accepted as the simplest and the most effective method from an industrial perspective, because this process makes it possible to fabricate high performance polymer nanocomposites at low process costs, and facilitates commercial scale-up [40,41,42,43,44,45,46,47,48,49]. Furthermore, the combination of a small quantity of CNTs with thermotropic LCPs provides attractive possibilities for improving the physical properties of polymer nanocomposites using a cost-effective method.

This paper focused on the fabrication and characterization of thermotropic liquid crystal copolyester (TLCP) nanocomposites reinforced with a very small quantity of CNTs using simple melt blending in a twin-screw extruder to create high performance composite materials with low processing cost. The effects of the unique characteristics of CNTs on the physical properties of TLCP/CNT nanocomposites are discussed. The attempt to disperse CNTs in TLCP matrix and to fabricate TLCP/CNT nanocomposites by simple melt compounding has been rarely investigated to date, and the effects of CNTs on the mechanical, rheological, and thermal properties of TLCP/CNT nanocomposites have not yet been reported in the literature. Thus, it is expected that this paper will be helpful in providing the preliminary understanding on the physical properties of TLCP nanocomposites reinforced with a very small quantity of CNTs. In addition, this paper suggests a simple and cost-effective method that will facilitate the industrial realization of TLCP nanocomposite with enhanced physical properties, providing a design guide of TLCP/CNT nanocomposites with a great industrial use potential. The exhaustive description of all LCP composites containing CNTs is beyond of the scope of this paper, and for this readers are referred to recent, excellent review papers [50,51,52].

## 2. Results and Discussion

### 2.1. Acid oxidation of carbon nanotubes

**Figure 1:**
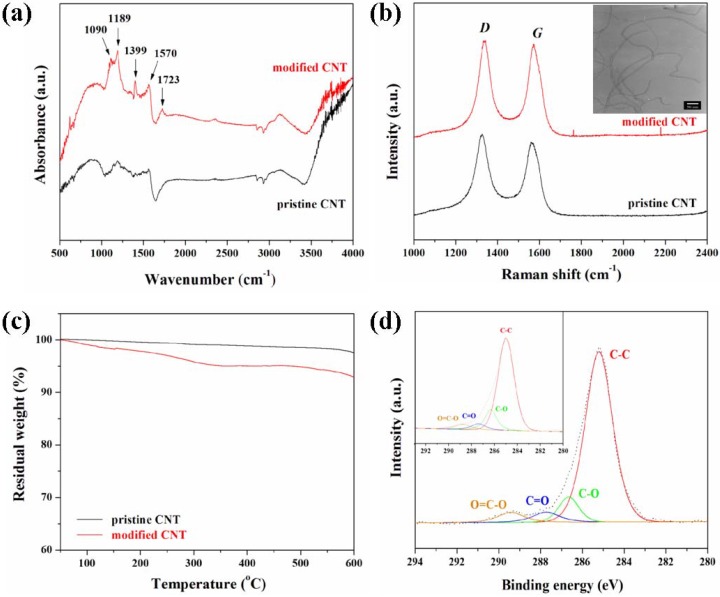
Characterization of modified CNT and TLCP nanocomposites. (a) FT-IR spectra of pristine and modified CNT. (b) Raman spectra of pristine and modified CNT. The decrease in the relative intensity ratio of *G*-band to *D*-band (*I*_G_/*I*_D_) of modified CNT indicates the increase in the degree of disorder and the presence of the defects on the surface of modified CNT by chemical modification. The inset shows TEM image of modified CNT. (c) TGA thermograms of pristine and modified CNT under N_2_. (d) High resolution C_1s_ XPS spectra of TLCP nanocomposite containing 1.5 wt% of modified CNT. The inset shows high resolution C_1s_ XPS spectra of neat TLCP. The shifting of characteristic peaks indicates the interactions between modified CNT and TLCP macromolecular chains in TLCP nanocomposites. Reproduced with permission from Ref. [43]. © 2008 Elsevier Ltd.

For polymer nanocomposites containing CNTs, homogeneous dispersion of CNTs in the polymer matrix and good interfacial adhesion between CNTs and the polymer matrix is required in order to overcome the limitations of efficient load transfer to polymers, playing a limited reinforcement role in polymer nanocomposites. In general, acid oxidation of CNTs is considered as an effective method to achieve the uniform dispersion of CNTs and their compatibility with the polymers, leading to the enhancement of the interfacial adhesion between CNTs and polymer matrix [32]. The carboxylic acid groups effectively introduced on the surface of CNTs increase the chemical affinity of the thus modified CNT with the polymer matrix. As shown in Figure 1(a), the characteristic peak at ~1,570 cm^−1^ was attributed to the IR-phonon mode of multi-walled CNTs [53]. For the modified CNTs, characteristic peaks were observed at 1,090, 1,189, 1,399, and 1,723 cm^−1^, corresponding to the stretching vibrations of the carboxylic acid groups [54]. Similar patterns observed in the Raman spectra of pristine and modified CNTs indicated that this chemical modification did not affect the graphite structure of CNTs (see Figure 1c). Two characteristic peaks observed at around 1,350 and 1,570 cm^−1^ can be termed as *D*-band and *G*-band, respectively [55,56].

The *G*-band, *i.e.,* the Raman-allowed phonon high-frequency mode, was related to the structural intensity of the *sp*^2^-hybridized carbon atoms of CNTs, and the *D*-band reflected the disorder-induced carbon atoms, resulting from the defects in CNTs and their ends, and its intensity decreases with the degree of the graphitization of CNTs. The intensity of *D*-band was slightly increased in the modified CNT and the relative *G*-band to *D*-band ratio (*I*_G_/*I*_D_) of the modified CNT was slightly increased as compared to pristine CNT, which was attributed to the increase in the degree of disorder and the presence of the defects on the surface of modified CNT as a result of the chemical modification. As shown in Figure 1(c), for the modified CNT, the first weight loss observed below 100 °C in the TGA thermograms was attributed to the loss of water molecules in the nanocomposite samples [57]. In addition, the further gradual decrease in the residual weight was attributed to organic decomposition of thermally unstable functional groups formed on the surface of modified CNT [58].

**Figure 2:**
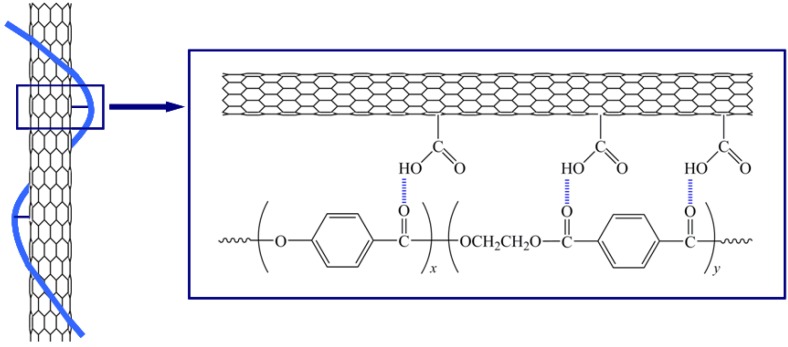
Schematic of possible interactions between modified CNT and TLCP though hydrogen bond formation.

This result demonstrates that carboxylic acid groups were effectively introduced on the surface of modified CNT via chemical modification. In general, CNTs often tend to bundle together and form agglomerations due to the intrinsic van der Waal attractions between the individual tubes in combination with their high aspect ratio and large surface area, thus leading to the obstruction of efficient load transfer to the polymer matrix [24,25]. The inset of Figure 1(b) shows that the modified CNT exhibits less entangled organization after chemical modification, instead of showing the agglomerates or bundles observed in pristine CNT. This feature makes it possible to enhance the dispersion of modified CNT relative to pristine CNT. Therefore, it is expected that the functional groups induced on the surface of CNT will be helpful for enhancing the dispersion state of CNT and the interfacial interaction in TLCP nanocomposites through hydrogen bonding formation, as illustrated in Figure 2. However, it was reported that there was a possibility to cause slight damage and a decreased length in the CNT structure induced by strong acid treatment, perhaps having a negative effect on the physical properties of the resulting nanocomposites [59]. In the XPS survey spectra, two characteristic peaks corresponding to C_1s_ and O_1s_ at around 285 and 523 eV were observed, which were assigned to the *sp*^2^ carbon and the ester-type groups with oxygen bonded to carbon [60]. High resolution C_1s_ XPS spectra of TLCP and TLCP nanocomposite containing 1.5 wt% of modified CNT are shown in Figure 1(d). The C_1s_ spectra of neat TLCP exhibited four characteristic peaks at 285.0, 286.5, 287.5, and 289.0 eV, corresponding to the C−C, C−O, C=O, and O=C−O groups, respectively. However, the C_1s_ spectra of TLCP nanocomposites shifted to higher binding energy, and also revealed the presence of four peaks, corresponding to the C−C (285.3 eV), C−O (286.8 eV), C=O (287.8 eV), and O=C−O (289.6 eV) groups, respectively. This slight shifting of the characteristic peaks to higher bonding energy for TLCP nanocomposites was attributed to some interactions of the functional groups induced on the surface of modified CNT via chemical modification with TLCP macromolecular chains in TLCP nanocomposites.

### 2.2. Thermal stability

**Figure 3:**
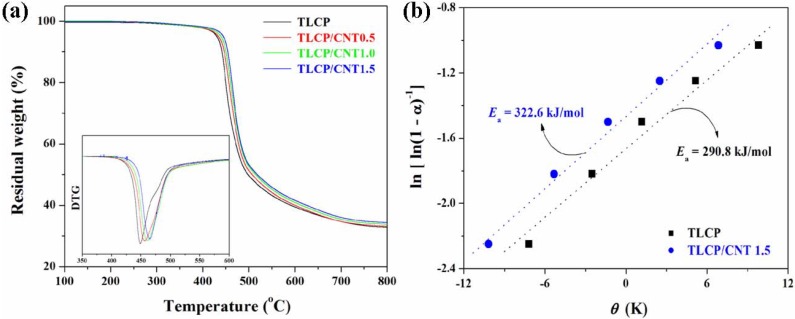
Thermal stability of TLCP nanocomposites. (a) TGA curves and (b) the plots of ln[ln(1-*α*)^−1^] versus *θ* for TLCP nanocomposites. By Eq. (1), the slope provides an estimate of activation energy (*E*_a_) for thermal decomposition of TLCP nanocomposites. Reproduced with permission from Ref. [43]. © 2008 Elsevier Ltd.

The thermal stability of polymer nanocomposites plays an important role in determining their working temperature limits and the environmental conditions for their use, which are related to their thermal decomposition temperature and decomposition rate. The patterns of TGA curves of TLCP nanocomposites are similar to those of pure TLCP, indicating that the thermal decomposition of TLCP nanocomposites must mostly stem from the TLCP. As shown in Figure 3, the incorporation of CNTs into TLCP matrix increased the thermal decomposition temperatures and the residual yields of TLCP nanocomposites. The presence of CNT could lead to the stabilization of TLCP matrix, resulting in the enhancement of the thermal stability of TLCP nanocomposites. The introduced CNT could induce protective barriers against thermal decomposition and retard the thermal decomposition of TLCP nanocomposites, resulting from the effective function of CNTs acting as physical barriers to hinder the transport of volatile decomposed products out of TLCP nanocomposites during thermal decomposition [61]. In addition, the incorporation of pristine and modified CNT into TLCP matrix increased the thermal stability of TLCP nanocomposites, and this enhancing effect was more significant in TLCP nanocomposites containing modified CNTs. For TLCP nanocomposites containing modified CNTs, good interfacial adhesion between modified CNT and TLCP matrix restricted the thermal motion of TLCP macromolecules [62], resulting in further enhancement of the thermal stability. TGA kinetic analysis was also conducted on TLCP nanocomposites to clarify the effect of CNT on thermal stability of TLCP nanocomposites. The decomposition temperatures and decomposition kinetic parameters such as the initial decomposition temperatures, the integral procedure decomposition temperature, the temperature at maximum rate of the weight loss, and the activation energy for thermal decomposition are commonly used to determine the thermal stability of polymers or composites. The activation energy for thermal decomposition (*E*_a_) of TLCP nanocomposites can be calculated from TGA thermograms by using the Horowits-Metzger integral kinetic method [63] as follows:

(1) $$\text{ln}\left[\text{ln}{\left(1-\alpha \right)}^{-1}\right]=\frac{E_{a}\theta }{RT_{dm}^{2}}$$

where *α* is the weigh loss; *E*_a_ is the activation energy for thermal decomposition; *T*_dm_ is the temperature at the maximum rate of weight loss; *θ* is the variable auxiliary temperature defined as *θ* = *T* – *T*_dm_, and *R* is the universal gas constant. The *E*_a_ value can be estimated from the slope of the plot of ln[ln(1 - *α*)^−1^] versus *θ* as shown in Figure 3(b). The *E*_a_ values of pure TLCP and TLCP/CNT nanocomposites were estimated to be 290.8, 293.7, 303.8, and 322.6 kJ/mol, respectively. As compared to pure TLCP, higher *E*_a_ value of TLCP nanocomposites suggested that they were more thermally stable than pure TLCP. In addition, TLCP nanocomposites containing modified CNT showed higher *E*_a_ values as compared to TLCP nanocomposites containing pristine CNT, suggesting that TLCP nanocomposites with more uniform dispersion of modified CNT were more thermally stable than those containing pristine CNT. This feature was also attributed to the interactions between modified CNT and TLCP matrix, which increase the activation of the thermal decomposition of TLCP matrix relative to pristine CNT. Similar observation has been reported that the enhanced thermal stability of CNT-reinforced poly(ethylene 2,6-naphthalate) (PEN) nanocomposites was associated with the increased interfacial interactions between modified CNT and PEN [41]. Because of excellent thermal conductivity of CNTs [64], the enhanced interfacial interactions between modified CNT and TLCP matrix resulted in the increased thermal conductivity of TLCP nanocomposites, leading to the improvement in the thermal stability.

### 2.3. Rheological behavior

**Figure 4:**
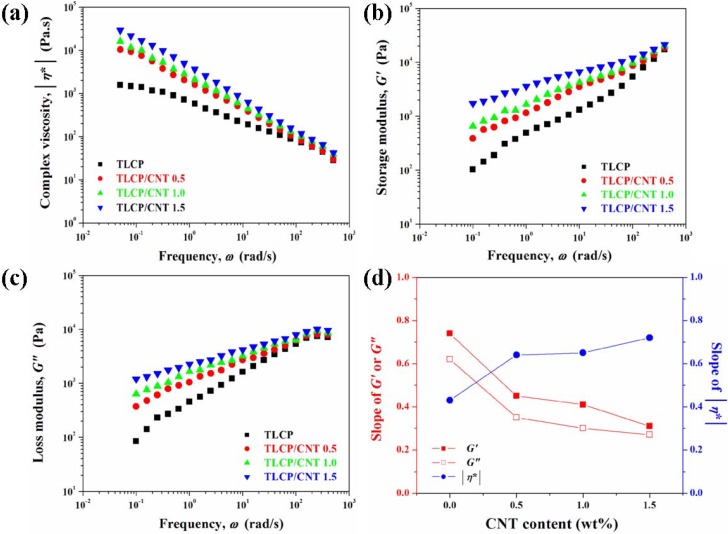
Rheological properties of TLCP nanocomposites. (a) Complex viscosity (|η^*^|), (b) storage modulus (*G*ʹ), and (c) loss modulus (*G*ʺ) of TLCP nanocomposites as a function of applied frequency. Reproduced with permission from Ref. [43]. © 2008 Elsevier Ltd. (d) Variations of slopes of *G*ʹ, *G*ʺ, and |η^*^| versus *ω* for TLCP nanocomposites with CNT content. Data from Ref. [43].

The rheological properties of TLCP nanocomposites are shown in Figure 4. The complex viscosities (|η^*^|) of pure TLCP and TLCP nanocomposites decreased with increasing frequency, indicating a non-Newtonian behavior over the whole frequency range studied. The shear thinning behavior of TLCP was attributed to the random orientation of rigid molecular chains during shearing force. The TLCP nanocomposites exhibited higher |η^*^| than that of pure TLCP at low frequency, indicating that the interconnected or network-like structures of CNTs were formed in TLCP nanocomposites because of the nanotube-nanotube or nanotube-polymer interactions. In addition, TLCP nanocomposites exhibited strong shear thinning behavior, resulting from the break-down of these structures with increasing frequency. As shown in Figure 4(a), the enhancing effect of |η^*^| of TLCP nanocomposites with increasing CNT content was more pronounced at low frequency than that at high frequency. The irregular decrease in the |η^*^| with increasing frequency indicated the pseudo-plastic characteristics of TLCP nanocomposites due to random orientation and entangled molecules in TLCP nanocomposites. As compared to TLCP nanocomposites containing pristine CNT, the complex viscosity of TLCP nanocomposites containing modified CNT was further increased due to the increased interactions between modified CNT and TLCP matrix. Furthermore, TLCP nanocomposites containing modified CNT exhibited higher |η^*^| and more distinct shear thinning behavior over the whole frequency range studied relative to pure TLCP and pristine CNT, suggesting either better dispersion of modified CNT or stronger nanotube-polymer interactions. Similar observation has been reported that higher viscosity and more distinct shear thinning behavior were indicative of stronger interfacial interactions in the CNT/epoxy nanocomposites with more uniform dispersion of CNTs [65]. The increase in the |η^*^| of TLCP nanocomposites was closely related to the large increase in the storage modulus. The shear thinning exponent (*n*) of TLCP nanocomposites can be estimated from the relationship of |η^*^| ≈ *ω* ^n^ [66]. There is a significant dependence of shear thinning behavior of TLCP nanocomposites on the presence of CNT. The shear thinning exponents of TLCP nanocomposites slightly decreased by incorporating pristine and modified CNTs, and this effect was more pronounced in the case of modified CNT. As shown in Figure 4(d), the *n* values of TLCP nanocomposites significantly decreased with the introduction of modified CNT, which may be related to the enhancement of the interfacial interactions between modified CNT and TLCP matrix as well as the uniform dispersion of modified CNT. In addition, TLCP composites containing pristine and modified CNTs exhibited slightly different values of shear thinning exponent even at the same CNT content, indicating the influence of the modification on the extent of CNT dispersion. In this study, TLCP nanocomposites exhibited the relationship between shear thinning behavior and mechanical properties: the more the shear thinning behavior, the better reinforcing effect on the mechanical properties of TLCP nanocomposites. Wagner and Reisinger [67] reported that the tensile modulus of poly(butylene terephthalate) nanocomposites containing modified clay prepared via melt compounding was significantly enhanced with lower values of the shear thinning exponent. This result will be described in the following discussion of the mechanical properties of TLCP nanocomposites.

The storage modulus (*G*ʹ) and loss modulus (*G*ʺ) of TLCP nanocomposites as a function of frequency are shown in Figure 4(b) and Figure 4(c). The values of *G*ʹ and *G*ʺ of TLCP nanocomposites significantly increased with increasing frequency and CNT content, and this enhancing effect was more pronounced at low frequency region. This feature is similar to the relaxation behavior of typical filled-polymer composite system [68,69]: if polymer chains are fully relaxed and exhibit a characteristic homopolymer-like terminal behavior, the flow curves of polymers can be expressed by a power law of *G*ʹ ∝ *ω*^2^ and *G*ʺ ∝ *ω*. Krisnamoorti *et al*. [70,71] reported that the slopes of *G*ʹ(*ω*) and *G*ʺ(*ω*) for layered silicate and polymer nanocomposite system were much smaller than 2 and 1, respectively, and they suggested that large deviations in the presence of a small quantity of layered silicate resulted from the formation of a network structure in polymer molten state. As shown in Figure 4(d), the variations of terminal zone slopes of TLCP nanocomposites indicated the non-terminal behavior with the power law dependence for *G*ʹ(*ω*) and *G*ʺ(*ω*) of TLCP nanocomposites. Similar non-terminal low-frequency behaviors have been also observed in the ordered block copolymers and the smectic liquid-crystalline small molecules [72,73]. The decrease in the slope of for TLCP nanocomposites with the introduction of CNT can be explained by the fact that the nanotube-nanotube or nanotube-polymer interactions can induce the formation of the interconnected or network-like structures, leading to the pseudo solid-like behavior more elasticity of TLCP nanocomposites relative to TLCP matrix. As the applied frequency increased, the interconnected or network-like structures were collapsed by high levels of shear force and TLCP nanocomposites exhibited almost similar or slightly higher *G*ʹ and *G*ʺ values than that of pure TLCP at high frequency region. As compared to pure TLCP and TLCP nanocomposites containing pristine CNT, higher values of *G*ʹ and *G*ʺ of TLCP nanocomposites containing modified CNT also suggested the increased interactions between modified CNT and TLCP matrix.

### 2.4. Morphology

**Figure 5:**
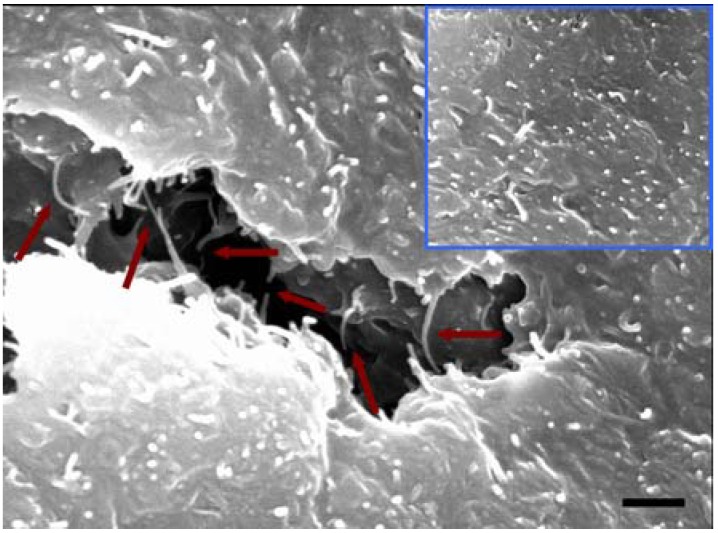
SEM micrograph of the fracture surface for TLCP nanocomposites containing 1.5 wt% of modified CNT (Scale bar: 500 nm). The inset show uniform dispersion of CNT in TLCP matrix. The arrow indicates that CNT were to be broken with their ends still embedded in TLCP matrix or they were bridging the local microcracks in TLCP nanocomposites, suggesting good wetting with TLCP matrix and enhanced adhesion between them, thus being favorable to efficient load transfer from TLCP to CNT. Reproduced with permission from Ref. [43]. © 2008 Elsevier Ltd.

The morphologies of TLCP/CNT nanocomposites are shown in Figure 5. In general, the drawbacks related to the uniform dispersion of CNT in the polymer matrix were caused by intrinsic van der Walls attractions between the individual nanotubes in combination with high aspect ratio and large surface area. This feature could make it difficult for CNT to disperse well in the polymer matrix and lead to more aggregated bundles of CNT, causing severe stress concentration phenomena and preventing efficient load transfer to the polymer matrix, thus resulting in some deterioration of the overall performance of the CNT/polymer nanocomposites. In this study, a chemical modification was performed in order to achieve both uniform dispersion of CNT in TLCP matrix and a stronger interfacial adhesion between CNT and TLCP. After chemical modification, CNT exhibited less entangled organization due to the functional groups formed on the nanotube surfaces instead of showing more agglomerated structures or bundles observed in the pristine CNT.

The modified CNT was, on a large scale, dispersed well in TLCP/CNT nanocomposites. Morphological observation was explained by the fact that CNT stabilized its dispersion by good interactions with TLCP, resulting from the interfacial interactions of –COOH groups of CNT with C=O groups of TLCP matrix. This interfacial adhesion between CNT and TLCP plays an important role in improving the overall mechanical properties of TLCP/CNT nanocomposites. It can be seen that two ends of CNT were covered by TLCP matrix despite a few CNT being pulled out from TLCP matrix. Interestingly, some of CNTs were broken with their two ends still strongly embedded in TLCP matrix, and bridging the local microcracks, which may delay the failure of polymer nanocomposites [74]. This result indicates that CNT has a good wetting with TLCP matrix, suggesting the existence of strong interactions between them. Similar observation has been reported that the presence of the fractured nanotubes, along with the polymer matrix still adhered to the fractured tubes matrix in terms of a crack interacting with the nanotube reinforcement significantly increased the mechanical properties of CNT/polystyrene nanocomposites [75]. For TLCP/CNT nanocomposites, the presence of functional groups on the surfaces of CNT resulted in the interfacial interaction between CNT and TLCP: the hydrogen atoms at –COOH groups of CNT may form hydrogen boding with C=O groups of TLCP molecular chains (Figure 2). Similar effect has been reported in CNT/polyamide (PA) nanocomposite systems that chemically modified CNT was effectively dispersed in PA matrix, because the functional groups of CNT probably have reacted with PA molecular chains during melt-mixing process, resulting in better dispersion state and stronger interfacial adhesion [76]. This result can be considered as the evidence for efficient load transfer from TLCP matrix to CNT during tensile testing, leading to the reinforcing effect on the overall mechanical properties of TLCP/CNT nanocomposites.

### 2.5. Mechanical behavior

**Figure 6:**
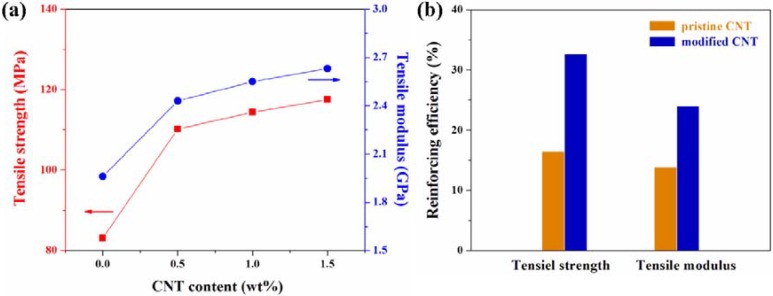
(a) Mechanical properties of TLCP nanocomposites with the CNT content. (b)Reinforcing efficiency of pristine and modified CNTs on mechanical properties of TLCP nanocomposites containing 0.5 wt% of CNT content. Reinforcing efficiency (%) = [(*M*_c_ – *M*_m_)/ *M*_m_] × 100, where *M*_c_ and *M*_m_ represent the mechanical properties, such as tensile strength and tensile modulus, of TLCP nanocomposites and TLCP, respectively. Data from Ref. [43].

For TLCP nanocomposites, there is a significant dependence of the mechanical properties on CNT content. The incorporation of CNT can substantially improve the mechanical properties of TLCP nanocomposites due to the nanoreinforcing effect of CNT with high aspect ratio and relatively uniform dispersion in TLCP matrix, and this enhancing effect was more pronounced at lower CNT content as compared to that at higher CNT content. At lower CNT contents, the incorporated CNTs were, on a large scale, uniformly dispersed in TLCP matrix, despite some entangled CNT or bundles. However, as compared to those at low CNT content, the mechanical properties of TLCP nanocomposites was not as significantly increased at higher CNT content as expected. Less uniformly dispersed and more entangled bundles of pristine CNT were formed in TLCP matrix at higher CNT content, which may be the cause of the stress concentration. This feature can be explained by the characteristics of CNTs that tend to bundle together due to the intrinsic van der Waals attractions in combination with high aspect ratio and large surface area [24,25] and can lead to some agglomeration, causing stress concentration phenomenon and preventing efficient load transfer from the reinforcing phase to the polymer matrix phase [26,27,28]. For PEN nanocomposites containing pristine CNT, the dependence of their mechanical propertieson CNT content has been also reported by Kim *et al*. [46,47,48,49]. Thus, for achieving further enhanced mechanical properties of TLCP nanocomposites, the enhancement of both uniform dispersion of nanotubes and the interfacial adhesion between nanotubes and polymer matrix should be required via functionalization of CNT. The mechanical properties of TLCP nanocomposites containing modified CNT are shown in Figure 6. The tensile strength and modulus of TLCP nanocomposites were substantially improved with the introduction of modified CNT by efficiently transferring load from the polymers to the nanotubes, and this enhancing effect was more pronounced with lower CNT content. Higher mechanical properties of TLCP/CNT nanocomposites was attributed to good interfacial adhesion between CNT and TLCP, suggesting that functional groups on modified CNT surface are helpful for improving the interfacial interaction with TLCP, thus being favorable to more effective load transfer from TLCP matrix to CNT. Thus, the improvement in homogeneous dispersion and interfacial adhesion in TLCP/CNT nanocomposites, resulting from strong interactions between modified CNT and TLCP, can lead to the substantial enhancement of mechanical properties of TLCP nanocomposites.

For characterizing the effect of modified CNT on the mechanical properties of TLCP nanocomposites, it is also very instructive to compare the reinforcing efficiency of pristine and modified CNT for a given content in TLCP nanocomposites. In this paper, the reinforcing efficiency of CNT can be defined as the normalized mechanical properties of TLCP nanocomposites with respect to those of pure TLCP. The enhancing effect of the mechanical properties by incorporating CNT was more pronounced in TLCP nanocomposites containing modified CNT than in the case of pristine CNT, indicating that the incorporation of modified CNT into TLCP matrix was more effective in improving the mechanical properties of TLCP nanocomposites. The incorporation of modified CNT containing functional groups effectively induced via chemical modification into TLCP matrix resulted in the increased interfacial adhesion between CNT and TLCP matrix, thus being favorable to more efficient load transfer form the polymers to the nanotubes. Therefore, the enhanced interfacial adhesion between modified CNT and TLCP matrix as well as uniform dispersion of modified CNT in TLCP matrix results in significant improvement in the overall mechanical properties of TLCP nanocomposites.

**Figure 7:**
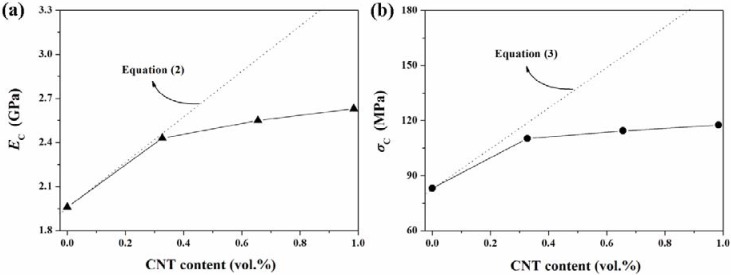
Comparative study for the mechanical properties of TLCP nanocomposites. Theoretically predicted values and the experimental results for (a) the nanocomposite modulus (*E*_C_) and (b) the nanocomposite strength (*σ*_C_) of TLCP nanocomposites. Dotted lines represent the theoretically predicted values based on the Equations (2) and (3). Reproduced with permission from Ref. [43]. © 2008 Elsevier Ltd.

A comparative study of the experimental results with the values predicted from the theoretical models is also very instructive to characterize the effect of CNT on the mechanical properties of TLCP/CNT nanocomposites. It is known that the modified Halpin-Tsai equation has been used to predict the modulus of CNT-filled polymer nanocomposites [77,78]. By assuming the random-oriented discontinuous distribution of modified CNT in TLCP matrix, the modified Halpin-Tsai equation can be expressed as follows [77,78,79,80]:

(2) $$\begin{array}{l} E_{C}=\left[\left(\frac{3}{8}\right)\left(\frac{1+2\left(l_{CNT}/d_{CNT}\right)\eta_{L}V_{CNT}}{1-\eta_{L}V_{CNT}}\right)+\left(\frac{5}{8}\right)\left(\frac{1+2\eta_{T}V_{CNT}}{1-\eta_{T}V_{CNT}}\right)\right]E_{TLCP}\\ \eta_{L}=\frac{\left(E_{CNT}/E_{TLCP}\right)-1}{\left(E_{CNT}/E_{TLCP}\right)+2\left(l_{CNT}/d_{CNT}\right)}\;\;and\;\;\eta_{T}=\frac{\left(E_{CNT}/E_{TLCP}\right)-1}{\left(E_{CNT}/E_{TLCP}\right)+2} \end{array}$$

where *E*_C_, *E*_TLCP_, and *E*_CNT_ are the tensile modulus of TLCP nanocomposites, TLCP, and CNT, respectively; *l*_CNT_/*d*_CNT_ is the ratio of length to diameter for CNT, and *V*_CNT_ is the volume fraction of CNT in the nanocomposites. To fit the Equation (2) to experimental results for TLCP nanocomposites, the weight fraction was transformed to the volume fraction, taking the densities of TLCP (1.41 g/cm^3^) [81] and perfectly graphitized CNT (2.16 g/cm^3^) [77,78]. The theoretically predicted values of TLCP nanocomposite modulus can be estimated assuming the aspect ratio of ~1,000 and *E*_CNT_ of ~450 GPa. The *E*_CNT_ values used in this paper represents a mid-range value in the modulus ranges of CNT previously measured [82]. The tensile strength of TLCP nanocomposites can be determined from the following equation [83]:

(3) $$\sigma_{C}=\sigma_{CNT}V_{CNT}+\sigma_{TLCP}V_{TLCP}$$

where *σ*_C_, *σ*_CNT_, and *σ*_TLCP_ are the tensile strength of the nanocomposites, CNT, and TLCP, respectively. The theoretically predicted values of TLCP nanocomposite strength can be estimated based on the *σ*_CNT_ values of ~11 GPa based on previous literature [84]. The theoretically predicted values and experimental data for TLCP nanocomposites modulus and strength are compared in Figure 7. At lower CNT content, the experimental results for mechanical properties of TLCP nanocomposites were fitted well with the theoretical equations, while the large deviations were observed at higher CNT content as compared to the theoretically predicted values. The nanoreinforcing effect of CNT on the mechanical properties of TLCP nanocomposites appeared to be more pronounced at lower CNT content than at higher CNT content. This result suggested that the interfacial interaction was more effective in the enhancement of mechanical properties of TLCP nanocomposites at lower CNT content than at higher CNT content. Kim *et al*. [41] reported that for modified CNT/PEN nanocomposite system, the interfacial interactions were more effective in improving the mechanical properties of modified CNT/PEN nanocomposites at lower CNT concentration than at higher CNT concentration. Thus, the synergistic effect of the increased interfacial interactions between modified CNT and TLCP matrix in combination with uniform dispersion of modified CNT was more effective at lower CNT content for enhancing the overall mechanical properties of TLCP nanocomposites.

However, the experimental results for mechanical properties of TLCP nanocomposites were lower than the theoretically predicted values, as shown in Figure 7. This result may be explained by the curvature of CNTs in TLCP nanocomposites: some CNTs embedded in TLCP matrix often exhibit curved morphology, and not a straight one (see Figure 5) and this curvature feature of CNT in TLCP matrix has a possibility to decrease the nanoreinforcing effect of CNT in TLCP nanocomposites, in comparison with the theoretical reinforcement provided by straight inclusions. Fisher *et al*. [85,86] have developed a model combining finite element results and micromechanical methods to determine the effective reinforcing modulus of wavy embedded CNT. They found that even slight curvature of CNT significantly reduced the effective reinforcement compared with straight CNT, and suggested that this feature may be an additional mechanism to limit the reinforcing effect of CNT on the mechanical properties of CNT/polymer nanocomposites, leading to the nanocomposite modulus and strength that were less than predicted by traditional theoretical models [85,86]. In addition, the orientation of CNT was an important factor in determining the mechanical properties of polymer nanocomposites, and the curvature morphology of CNTs was also related to the orientation of CNT in polymer nanocomposites. Gorga and Cohen [87] reported that aligned non-entangled CNTs should disperse and orient more readily in the polymer nanocomposites, thus improving their mechanical properties more significantly with optimal processing conditions. The nanoreinforcing effect of CNT will be more effective in improving the mechanical properties of TLCP nanocomposites when the introduced CNT exhibit straight morphology within TLCP matrix and they are preferentially aligned along their axial direction. Thus, the overall mechanical properties of TLCP nanocomposites is expect to be further improved by optimizing the unique geometric feature and alignment of CNT in TLCP matrix as well as the combination of the enhanced interfacial adhesion between CNT and TLCP matrix with the good dispersion of CNT in TLCP matrix during the melt processing.

## 3. Experimental Section

### 3.1. Materials

TLCP used was a flexible semi-aromatic copolyester synthesized from poly(*p*-hydroxybenzoate) (PHB) and poly(ethylene terephthalate) (PET) with a molar ratio of 80:20, purchased from Unitika Co. Ltd, Japan. The nanotubes used were multi-walled CNT (degree of purity >95%) synthesized by a thermal chemical vapor deposition process, purchased from Iljin Nanotech Co., Korea. The diameter and length of CNT were in the range of 10~30 nm and 10~50 μm, respectively. Concentrated nitric acid (HNO_3_, 68%) and sulfuric acid (H_2_SO_4_, 98%) were purchased from Sigma Aldrich Co., and they used as received without further purification.

### 3.2. Fabrication of TLCP/CNT nanocomposites

Pristine CNT was modified by the following steps: CNT was added to the mixture of concentrated HNO_3_ and H_2_SO_4_ with a volumetric ratio of 1:3 and this mixture was sonicated at 80 °C for 4 h to create the carboxylic acid groups on the nanotube surface. This mixture was diluted with excess deionized water and then vacuum-filtered through 0.22 μm milipore PTFE membranes until the pH of the filtrate reached approximately 7. The filtrate solid was dried *in vacuo* at 100 °C for 24 h, yielding the modified CNT. The carboxylic acid groups on the surface of CNT were effectively induced via this chemical modification [32] to increase the chemical affinity of CNT with TLCP, yet to decrease the π–π stacking effect among the aromatic rings of the nanotubes, which often leads to the formation of their agglomeration [88]. All materials were dried at 120 °C *in vacuo* for at 24 h before use, to minimize the effects of moisture. The TLCP nanocomposites were prepared by a melt blending in a Haake rheometer (Haake Technik GmbH, Germany) equipped with a twin-screw (intermeshing co-rotating type). The temperature of the heating zone, from the hopper to the die, was set to 290, 300, 305, and 295 °C, and the screw speed was fixed at 40 rpm. Prior to melt blending, TLCP and CNT were physically premixed before being fed into hopper of the extruder to achieve better dispersion of CNT with TLCP matrix. For the fabrication of TLC nanocomposites, TLCP was melt blended with the addition of CNT content, specified as 0.5, 1.0, and 1.5 wt% in TLCP matrix, respectively. Upon completion of melt blending, the extruded strands were allowed to cool in water-bath, and then cut into pellets with constant diameter and length using a rate-controlled PP1 pelletizer (Haake Technik GmbH).

### 3.3. Characterization

The chemical structure of CNT was characterized by means of room temperature FT-IR measurements in the range of 400~4,000 cm^−1^ recorded using a Magna-IR 550 spectrometer (Nicolet Co.). The change in the structure of CNT by chemical modification was characterized by means of a JASCO NRS-3100 Raman spectrometer (JASCO Inc.) equipped with a 532 nm diode laser, and the laser beam with a nominal power of 30 mW was focused to a spot size of 5 μm diameter. Morphologies of CNT and TLCP nanocomposites were observed using a JEOL 2000 FX TEM and a JEOL JSM-6340F SEM. Rheological properties of TLCP nanocomposites were performed on an ARES (Advanced Rheometric Expansion System) rheometer (Rheometric Scientific, Inc.) in oscillation mode with the parallel-plate geometry using the plate diameter of 25 mm and the plate gap setting of ~1 mm at 305 °C, by applying a time-dependent strain, *γ*(*t*) = *γ*_0_sin(*ωt*) and measuring resultant shear stress, *γ*(*t*) = *γ*_0_ [*G*ʹsin(*ωt*) + *G*ʺcos(*ωt*)], where *G*ʹ and *G*ʺ are storage and loss moduli, respectively. The frequency ranges were varied between 0.05 and 450 rad/s, and the strain amplitude was applied to be within the linear viscoelastic ranges. The mechanical properties of TLCP nanocomposites were measured using an Instron 4465 testing machine, according to the procedures in the ASTM D 638 standard. Thermogravimertic analysis of TLCP nanocomposite were performed with a TA instrument SDF 2960 TGA over the temperature rage of 30~800 °C at a heating rate of 10 °C/min under N_2_.

## 4. Conclusions

This paper focused on the fabrication and characterization of thermotropic liquid crystal polymer (TLCP) nanocomposites reinforced with a very small quantity of modified carbon nanotube (CNT). The TLCP nanocomposites exhibited higher complex viscosity and more distinct shear thinning behavior than pure TLCP, resulting from strong interactions between CNT and TLCP matrix, which significantly influenced the relaxation behavior of polymer chains in the nanocomposites. The extent of the increase in the complex viscosity, storage modulus, and loss modulus of TLCP nanocomposites by the presence of modified CNT was more pronounced at low frequency as compared to those at high frequency. The decrease in the slopes of storage and loss moduli for TLCP/CNT nanocomposites with the introduction of modified CNT was attributed to the formation of the interconnected or network-like structures via the nanotube-nanotube and polymer-nanotube interactions, resulting in the pseudo solid-like behavior of TLCP nanocomposites. The incorporated CNT has a significant effect on the enhancement of thermal stability by acting as effective physical barriers against the thermal decomposition in TLCP nanocomposites. The improvement in the mechanical properties of TLCP nanocomposites resulted from the enhanced interfacial adhesion between modified CNT and TLCP as well as uniform dispersion of modified CNT in TLCP matrix, and their synergistic effect combining good interfacial interaction and uniform dispersion was more effective at lower CNT content relative to higher CNT content. This study demonstrates that the optimization of straight morphology and preferential alignment of CNT during melt processing as well as the enhanced interfacial adhesion between CNT and TLCP and uniform dispersion of CNT in TLCP matrix plays a key role in further improving the overall mechanical properties of TLCP nanocomposites.
